# Severe Concomitant *Physaloptera* sp., *Dirofilaria immitis*, *Toxocara cati*, *Dipylidium caninum*, *Ancylostoma* sp. and *Taenia taeniaeformis* Infection in a Cat

**DOI:** 10.3390/pathogens10020109

**Published:** 2021-01-22

**Authors:** Jose Cesar Menk P. Lima, Fabio Del Piero

**Affiliations:** Louisiana Animal Disease Diagnostic Laboratory (LADDL), Pathobiological Sciences Department, School of Veterinary Medicine, Louisiana State University, Baton Rouge, LA 70803, USA

**Keywords:** *Physaloptera*, *Dirofilaria*, parasitosis, cat

## Abstract

Here we describe an unusual and severe mixed parasitic infection in a cat that died during routine surgery. Gastric *Physaloptera* sp., cardiac *Dirofilaria immitis*, and intestinal *Toxocara cati*, *Dipylidium caninum*, *Ancylostoma* sp. and *Taenia taeniaeformis* were observed. Histologic lesions included chronic proliferative pulmonary endarteritis, mild increase of mucosal intestinal white cells, and terminal aspiration of gastric content. The severe dirofilariasis may have contributed to this patient death during anesthesia.

## 1. Introduction

Domestic cats (*Felis catus*) can be infected by diverse intestinal parasites, some of which have zoonotic potential. Most gastrointestinal parasites occurring in cats have a higher prevalence in younger individuals and cats housed in crowded conditions, such as shelters [1]. Several factors can influence the prevalence of different parasites, including the host population and its geographic location, as well as the sensitivity of tests used [1].

In large studies carried out on various free-roaming and sheltered feline populations across different regions of North America over the past few years, parasites such as *Toxocara cati*, *Ancylostoma* sp. and *Cryptosporidium* sp. are among the most frequently reported [2,3,4]. In one of these studies, nematodes of the genus *Physaloptera* were reported in 2.2% of the total sampled cat population [2].

*Physaloptera* is a genus of nematodes found in the stomachs and occasionally in the duodenum of cats and dogs, as well as a wide range of other mammals, birds and reptiles worldwide [5,6,7]. In North America, these parasites have been described in dogs, coyotes, raccoons, wolves, foxes, cats, bobcats, and, recently, in a bobwhite quail [5,6]. These gastrointestinal parasites are transmitted via an indirect life cycle and utilize a variety of arthropod intermediate hosts, including flour beetles, cockroaches, and crickets [1,6]. *Physaloptera* spp. also have several paratenic hosts, including amphibians, reptiles, and mammals. Cats may become infected after feeding on intermediate hosts infected by *Physaloptera* spp. larvae or by predation of paratenic hosts, such as mice, that have previously fed on an intermediate host [1,5]. Adult worms measure 1–6 cm depending on species, and the prepatent period is 8–10 weeks [8].

After ingestion, these nematodes firmly attach to the gastric and duodenal mucosa, where they start feeding on blood [6]. The diagnosis of physalopterosis is based on observation of clinical signs such as chronic intermittent vomiting, visualization of the parasite in vomited debris or during endoscopy, and by the observation of parasite eggs in the feces by flotation [6].

The aim of this report is to describe an uncommon and very complex case of massive *Physaloptera* sp. infection associated with other helminth infections, including possible fatal dirofilariasis, in a cat.

## 2. Case Report

A seven-month-old female spayed domestic shorthair feline from a south Louisiana animal shelter was received for necropsy at the Louisiana Animal Disease and Diagnostic Laboratory (LADDL) with a history of having died at the conclusion of an ovariohysterectomy procedure, during which administration of atropine through an endotracheal tube and CPR could not resuscitate the animal. The presence of cestode proglottids around the anus was reported by the veterinary surgeon.

**Figure 1:**
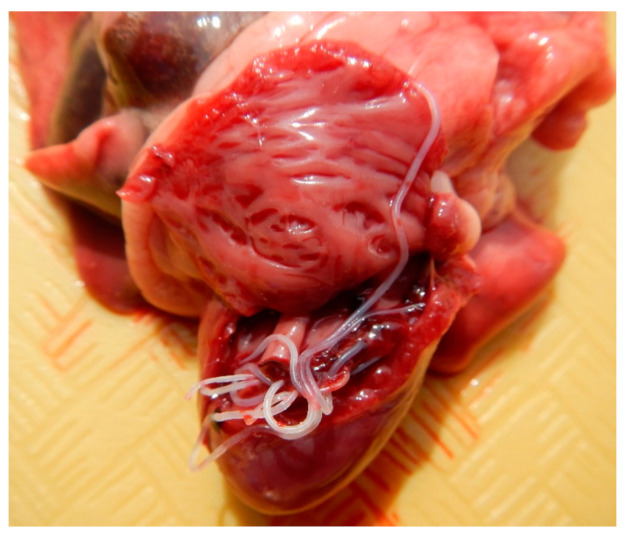
Heart containing several *Dirofilaria immitis* adult nematodes within the right cardiac ventricle.

**Figure 2:**
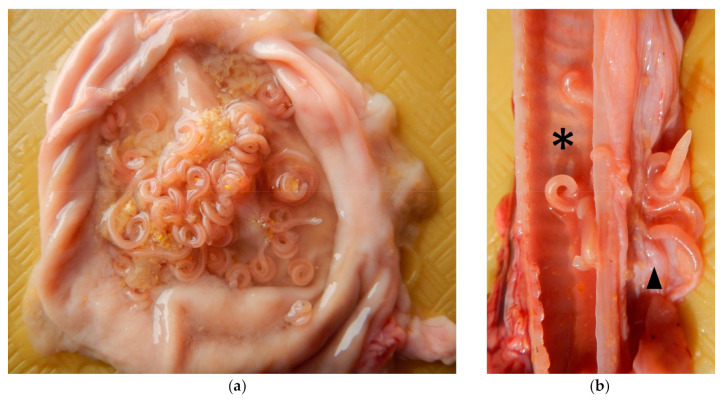
(**a**) Stomach containing numerous *Physaloptera* sp. mixed with stomach contents. (**b**). Trachea (asterisk) and esophagus (arrowhead) containing several *Physaloptera* sp.

**Figure 3:**
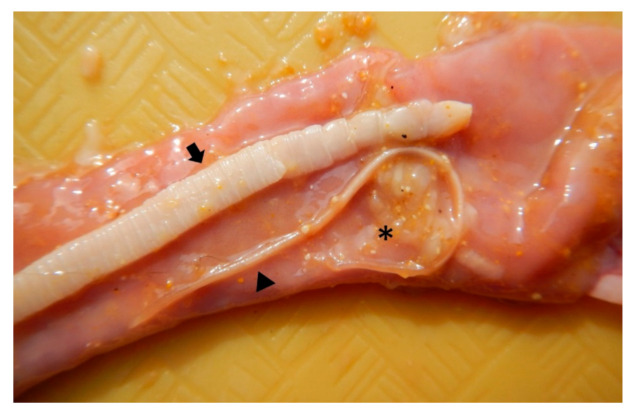
Small intestine containing a large tapeworm identified as *Taenia taeniaeformis* (arrow), a single *Toxocara cati* (arrowhead) and multiple *Dipylidium caninum* proglottids (asterisk).

On postmortem examination, the animal weighted 2.2 kg, with an adequate nutritional and muscular condition and minimal postmortem changes. There was a small amount of green, yellow, mucoid material in the nasal cavity and on the face around the mouth, indicating regurgitation. A 2 cm surgical incision was present on the ventral abdominal midline. Five ml of serosanguineous fluid were contained within the thoracic cavity. Within the right cardiac ventricle, extending within the pulmonary artery, there were four white nematodes, 4–6 cm in length, identified as *Dirofilaria immitis* (Figure 1). Multifocal green to brown areas were noted on the right cranial lung lobe, right middle lung lobe, right caudal lung lobe, left cranial lung lobe, and left caudal lung lobe. The oral cavity contained two 2.5 × 1 cm, off white and coiled nematodes identified as *Physaloptera* sp. Two similar nematodes were contained within the trachea, four were found in the esophagus and approximately 30 were within the stomach (Figure 2). In the jejunal lumen, there were five white, flat and segmented 3–4 cm long cestodes consistent with *Dipylidium caninum*, four white 5–6 cm long nematodes identified as *T. cati* and two white, flat, segmented 10–12 cm long cestodes identified as *Taenia taeniaeformis* (Figure 3). The omentum was diffusely pale. No other significant gross lesions were noted.

Five micron tissue sections from the lungs, heart, spleen, kidney, liver, bone marrow, stomach, intestine, brain, vessels, nerves, and ganglia were examined microscopically.

In the lungs, branches of the pulmonary artery had thickening of the tunica intima by abundant fibrous connective tissue with few lymphocytes and plasma cells, and formation of extensive frond-like proliferations into the lumen. Small amounts of cell debris mixed with eosinophilic hyaline material were contained in multiple bronchi, bronchioles, and alveoli, likely due to inhalation. There was mild to moderate congestion and edema with increased numbers of macrophages.

In the liver, there was severe diffuse hydropic degeneration of hepatocytes. The spleen had mild depletion of lymphocytes from lymphoid follicles and follicular hyalinosis, with extramedullary hematopoiesis.

In the jejunum, there was mild diffuse increase in numbers of proprial plasma cells and lymphocytes.

The specimens of *D. immitis*, *Physaloptera* sp., *T. cati*, *T. taeniaeformis*, and *D. caninum* were initially identified in situ based on descriptions by Baker [9]. Feces and one of the stomach parasites were submitted for routine fecal flotation and parasite identification, respectively. Eggs of *Ancylostoma* sp., *Physaloptera* sp. and *T. cati* were identified on centrifugal fecal flotation with sucrose solution [10] based on descriptions of the eggs by Baker [9], while the gastric parasites were directly identified as *Physaloptera* sp. [8,9,11].

## 3. Discussion

The most unusual feature of this clinical case is the severity of the mixed parasitic infection caused by several parasite species found in the digestive and cardiorespiratory tracts of the cat, mainly *Physaloptera* sp. found in the stomach and *D. immitis* within the right cardiac ventricle and the pulmonary artery. *Toxocara cati*, *D. caninum* and *T. taeniaeformis* were observed in the small intestine, while *Ancylostoma* sp. was detected only at fecal examination. 

*Toxocara cati* is among the most commonly detected parasites in cats according to several published surveys in North America [2,3,4]. This nematode infects felines via ingestion of infective eggs (direct transmission), transmammary transmission or ingestion of infected paratenic hosts such as rodents (indirect transmission). The majority of infections are acquired through the transmammary and paratenic host routes, and; therefore, lesions are usually restricted to the intestines, with clinical signs such as diarrhea, poor coat condition and failure to thrive. Adult nematodes are 3–10 cm in length and are found in the small intestine. Eggs are shed in the feces after a prepatent period of about eight weeks [8,12].

Cats acquire *D. caninum* infection through the ingestion of fleas infected with the larval stage (cysticercoid) of this cestode. Recent studies describe two different genotypes for this species of cestode, with different host associations in cats versus dogs involving a shorter prepatent period and longer lifespan depending on the final host species, while also suggesting the possible existence of two host associated *Dipylidium* species [13,14]. After ingestion of the infected flea by a cat, usually during grooming, there is a prepatent period of 34 days on average. Adult parasites are 20–50 cm in length and shed motile proglottids containing egg sacs in the feces, which may be observed in the perianal area. Adult tapeworms are of little pathogenic significance [8,12].

*Taenia taeniaeformis* (also called *Hydatigera taeniaeformis*) have rodents as intermediate hosts and these tapeworms are usually found in cats that actively hunt. After ingestion, there is a prepatent period of approximately six weeks; adults may be up to 70 cm in length, infections are usually subclinical and proglottids are shed in the feces [8,12].

*Ancylostoma* spp. is a common genus of hookworm that affects multiple animal species, and *Ancylostoma tubaeforme* is the most widely distributed hookworm in cats [2,12]. Cats can be infected via cutaneous penetration, ingestion of larvae, or ingestion of an infected paratenic host such as rodents. Adults can be found in the small intestine and measure 1–3 cm in length; eggs are shed in feces and larvae are infective to other felines and paratenic hosts, with a prepatent period of two to four weeks. Even though pathogenicity of these hookworms is considered low, heavy infections may lead to anemia, reduced growth and poor coat condition [8,12].

While the previous history of the cat in this case is incomplete, the body was received from an animal shelter facility in southern Louisiana, and the heavy, diverse parasitic burden is indicative of exposure to conditions that would favor these parasitic infections. Exposure to infectious parasitic stages and intermediate and paratenic hosts of different or multiple parasites is more likely to happen in free-roaming and shelter cats as a consequence of outdoor access, predation and scavenging, frequently paired with absence of veterinary care [2]. Thus, it is likely that this cat, other than being living in environments that would facilitate the infection by diverse parasites, did not receive proper veterinary care, which would also allow for the parasitic infections to go undetected.

It is important to underscore that in shelter situations where there is routine intake of free-roaming animals, with frequent overcrowding, parasite transmission between cats can be augmented. Strategies for prevention, which include testing cats for parasite infections and routine deworming, are of great importance during shelter stays and also after adoption, as many parasitic infections in cats have zoonotic potential and pose a constant risk for animal and human health [3,4].

While all the parasites observed in this case can cause varying degrees of disease in cats [2,12] and other domestic animals and several are a potential zoonotic threat, the heavy burden of *Physaloptera* sp. and *D. immitis* are considered to be the most important cause of disease in this animal.

Chronic intermittent vomiting is the most common clinical sign in cats affected by *Physaloptera* spp. [15]. While green to brown discoloration in the lung lobes was observed grossly, and extraneous material was observed microscopically within the airways, an inflammatory process did not accompany these findings. Therefore, the presence of *Physaloptera* sp. within the oral cavity and trachea at the time of necropsy is thought to represent trans-operatory or terminal aspiration. In case of trans-operatory aspiration, partial or total blockage of airways may be involved in the cause of death in this animal. *Physaloptera* spp. are also reported to cause gastric erosions after attaching to the gastric wall, often accompanied by marked catarrhal gastritis [6]. In this case; however, there were no gross or microscopic gastric lesions.

Severe proliferative villous endarteritis was observed in branches of the pulmonary artery and in the pulmonary parenchyma and are attributed to heartworms (*D. immitis*). It is important to note that despite being seven months old, this cat had already developed severe chronic heartworm disease, indicating that the infection occurred at a very young age. This brings to attention the importance of starting preventive care with macrocyclic lactones in kittens as early as eight weeks of age, as well as maintaining cats on preventives in areas where heartworm is also endemic in dogs [16]. Infection by four of these nematodes is considered a heavy burden for felines, as cats can have severe disease and sudden death in the presence of a small number of these parasites [17]. When compared to dogs, the development of *D. immitis* in cats takes longer, and most infections are amicrofilaraemic [17]. This is thought to be related to a long-term low and transient antibody response against L3 larvae despite a strong IgG antibody response during early infection, suggesting that the L3 stage can evade the host’s humoral immune response and develop into adults [18,19]. Microfilariae were not histologically observed within the tissues examined. Sudden death in cats with heartworm disease may be linked to systemic anaphylaxis characterized by increased dyspnea and reduced levels of blood O_2_ saturation, expired CO_2_, and systolic blood pressure [20]. The immune response is directed against the nematode antigens and *Wolbachia* endosymbionts that may be shed following the death of larvae or pre-adult *D. immitis* [17]. Given the possible fatal outcome, proper heartworm disease diagnosis prior to surgery should be considered for all cats not on regular preventive medication, particularly in hyperendemic areas.

Heartworm disease may have played an important role in this cat’s death. Heartworm-positive animals are considered at-risk patients for anesthesia; pulmonary embolism and congestive heart failure may arise from changes in cardiac output and pulmonary vascular resistance under general anesthesia [21]. To the best of our knowledge, there are no published studies focusing on response to anesthesia in felines with heartworm disease; however, it is reasonable to assume that the combination of general anesthesia and heartworm disease in this case may have led to a fatal outcome. It is uncertain; however, whether this was a result of acute anaphylaxis, alterations of vascular resistance enhanced by chronic proliferative endarteritis, or a combination of both.

While infection by multiple different parasites may be detrimental to systemic health, with the exception of *D. immitis,* the parasites identified are not usually listed as a cause of death on their own. Interestingly, the white cell population of this patient intestinal mucosa was only mildly increased. Regarding the evidence of pulmonary aspiration of gastric contents, the absence of inflammation related to the airways points towards a perimortem event rather than a chronic one. Nonetheless, partial obstruction of airways by aspirated debris would further restrict air intake and oxygenation.

## 4. Conclusions

This report highlights the extent and severity of parasitic infection that can be found in a single cat, bringing attention to the associated complications and importance of veterinary care and parasite control in felines, including sheltered animals, to prevent such an infection.

## Data Availability

No new data were created or analyzed in this study. Data sharing is not applicable to this article.
